# Is the operative delivery rate in low-risk women dependent on the level of birth care? A randomised controlled trial

**DOI:** 10.1111/j.1471-0528.2011.03043.x

**Published:** 2011-07-12

**Authors:** S Bernitz, R Rolland, E Blix, M Jacobsen, K Sjøborg, P Øian

**Affiliations:** a Department of Obstetrics and Gynaecology at Østfold Hospital TrustFredrikstad, Norway; b Department of Obstetrics and Gynaecology, Vestre Viken Hospital TrustDrammen, Norway; c Clinical Research Centre, University Hospital of North NorwayTromsø, Norway; d Faculty of Health Sciences, University of TromsøTromsø, Norway; e Department of Research, Østfold Hospital TrustFredrikstad, Norway; f Department of Clinical Medicine, University of OsloOslo, Norway; g Department of Obstetrics and Gynaecology, University Hospital of North NorwayTromsø, Norway

**Keywords:** Birth outcome, birth unit, low-risk birth, midwife-led unit

## Abstract

**Objective:**

To investigate possible differences in operative delivery rate among low-risk women, randomised to an alongside midwifery-led unit or to standard obstetric units within the same hospital.

**Design:**

Randomised controlled trial.

**Setting:**

Department of Obstetrics and Gynaecology, Østfold Hospital Trust, Tromsø, Norway.

**Population:**

A total of 1111 women assessed to be at low risk at onset of spontaneous labour.

**Methods:**

Randomisation into one of three birth units: the special unit; the normal unit; or the midwife-led unit.

**Main outcome measures:**

Total operative delivery rate, augmentation, pain relief, postpartum haemorrhage, sphincter injuries and intrapartum transfer, Apgar score <7 at 5 minutes, metabolic acidosis and transfer to neonatal intensive care unit.

**Results:**

There were no significant differences in total operative deliveries between the three units: 16.3% in the midwife-led unit; 18.0% in the normal unit; and 18.8% in the special unit. There were no significant differences in postpartum haemorrhage, sphincter injuries or in neonatal outcomes. There were statistically significant differences in augmentation (midwife-led unit versus normal unit RR 0.73, 95% CI 0.59–0.89; midwife-led unit versus special unit RR 0.69, 95% CI 0.56–0.86), in epidural analgesia (midwife-led unit versus normal unit RR 0.68, 95% CI 0.52–0.90; midwife-led unit versus special unit RR 0.64, 95% CI 0.47–0.86) and in acupuncture (midwife-led unit versus normal unit RR 1.45, 95% CI 1.25–1.69; midwife-led unit versus special unit RR 1.45, 95% CI 1.22–1.73).

**Conclusions:**

The level of birth care does not significantly affect the rate of operative deliveries in low-risk women without any expressed preference for level of birth care.

## Introduction

Over the last few decades there has been an increasing trend towards the centralisation of childbirth in larger clinics in developed countries. As the level of available obstetric technology increases, the use of this technology increases as well, leaving researchers to suggest that low-risk women may receive excess interventions.^1^–^4^ Intervention rates for low-risk births might be higher than necessary, and there are large variations in inter-unit comparisons.^5^ In their intrapartum care guidelines, the UK's National Institute for Health and Clinical Excellence concluded that if a low-risk woman plans to give birth in a midwife-led unit she will have a higher likelihood of a normal birth with less intervention.^6^

As a counterbalance towards the trend of increased perinatal intervention, low-risk birth units or birth centres have been established. Low-risk birth units can either be freestanding, i.e. localised away from a hospital, or sit alongside, i.e. integrated within a hospital. These units are most often midwife led. Transfer from a low-risk birth unit to a standard care birth unit or hospital is required if medical services are necessary.

Freestanding birth centres have been studied in different settings, concluding that birth centres are a safe alternative to hospital for low-risk women.^7^ It is also shown that general practitioners and midwives can identify a low-risk population that can deliver safely at maternity homes, with a low rate of operative deliveries and transfers.^8^ Freestanding, midwife-led birth centres report higher rates of normal births and lower rates of caesarean sections and episiotomies.^9^ Alongside birth units have also been studied widely. The Stockholm birth centre trial concludes that birth centre care is associated with less medical interventions, without statistically significant differences in health outcome.^10^

A Cochrane review on the topic concludes that an alternative birth setting versus conventional institutional birth setting is associated with reduced rates of medical interventions and increased maternal satisfaction, but states that there might be an increased risk for perinatal mortality.^11^ According to Gottvall et al.,^12^ there is no statistically significant difference in perinatal mortality between birth centres and standard care.

A systematic review on low-risk units concludes that birth centres can offer the possibility of accessible, appropriate and personal maternity care for women and their families, but points to a strong need for randomised trials.^13^ Hatem et al.^14^ conclude in a Cochrane review that women who had midwife-led care were less likely to experience operative delivery, with no statistically significant differences in fetal or neonatal death overall. Studies reporting results from low-risk units often include participants early in pregnancy.^10^,^15^–^18^ This implies that a certain number of women included do not fulfil the selection criteria for midwife-led units at onset of labour, and therefore do not attend these units at all in labour. Following the important principle of intention to treat, the participants are still analysed according to the group they were originally allocated to.Waldenstrøm and Nilsson^10^ state that among women randomised to the midwife-led unit, 34% were transferred antepartum and 16% were transferred intrapartum.

In this trial we wanted to study the effect of birth unit on birth outcome for low-risk women, and inclusion was therefore conducted at the onset of spontaneous labour. When searching for similar trials including women at onset of labour conducted in the last 20 years, only two randomised controlled trials were found: one from the USA and one from Hong Kong.^19^,^20^

Earlier data from several standard care obstetric departments in Norway show an operative delivery rate (caesareans, vacuum extractions and forceps deliveries) amongst low-risk women of ≥10%.^21^ At freestanding midwife-led units the operative delivery rate for the same group is approximately 5%.^22^

The aim of the present randomised controlled trial was to investigate if there were differences in operative delivery rates in low-risk women giving birth in an alongside, midwifery-led unit, compared with obstetric units. We hypothesised that it was possible to reduce the need for operative deliveries, with the same or better results for mother and child, if low-risk women were delivered in a separate low-risk unit.

## Methods

In 1999 the Norwegian Parliament decided to organise national birth care into three levels.

1. Departments of obstetric and gynaecology with more than 1500 births per year, providing all birth care services with obstetricians, paediatricians and anaesthesiologists on duty at all times, and with a neonatal intensive care unit.
2. Smaller obstetrical departments with 400–1500 births per year, providing low-risk birth care with obstetricians and anaesthesiologist on call.
3. Midwife-led maternity homes with 40–400 births per year, providing birth care for healthy women with expected normal births.

The Norwegian Parliament also advised obstetric departments to have low-risk units within hospitals.^23^ Therefore, the Department of Obstetrics and Gynaecology at Østfold Hospital Trust, with approximately 3000 births per year, was divided into three separate units, placed on separate floors, in 2004: The midwife-led unit (MU), the normal unit (NU) and the special unit (SU).

The MU is organised for low-risk women with expected normal births who want as little intervention as possible. Restrictive selection criteria must be fulfilled to attend this unit. No epidural is offered nor augmentation, unless required for the second phase of the second stage. If extended surveillance is needed or if the birth needs to be taken over by an obstetrician, the woman will be transferred to either the NU or the SU. Obstetricians are not present at the unit unless called on for a specific reason. The NU is organised for women with expected normal births. The unit has access to extended surveillance, epidural and operative vaginal delivery. It also provides room for women with elective caesareans and inductions after spontaneous rupture of membranes. If extended surveillance is necessary throughout the birth at the NU, a transfer to the SU is not required. The SU is organised for women who are in need of extended surveillance in the antenatal period, during labour and after birth.

Women expecting normal births may give birth at any of the three units, but at the MU only low-risk women are accepted. The MU has approximately 600 births annually, and the other two units have approximately 1200 births each. Each unit has its own separate staff, and midwives are responsible for all normal deliveries. All units provide both birth and postpartum care.

To explore our hypothesis a randomised trial was carried out. The primary outcome was operative delivery rates. Secondary outcomes were: augmentation of labour, pain relief, and postpartum haemorrhage, and neonatal outcomes measured by an Apgar score <7 at 5 minutes, metabolic acidosis defined as an umbilical artery pH <7.05 and BE (Base Excess) <−12 mmol/l,^24^ and transfers to the neonatal intensive care unit (NICU).

Information about the trial was sent to all women planning to give birth at Østfold Hospital Trust when being called for a routine ultrasound examination. At the routine ultrasound examination at 18–20 weeks of gestation, all women roughly suited for the trial received additional written and verbal information about the trial. If eligible for the trial and willing to participate, she was recruited for the trial. If she fulfilled the inclusion criteria at the onset of spontaneous labour, she was randomised to one of the three units.

The inclusion criteria for this study were similar to the selection criteria at the MU. Healthy, low-risk women without any disease known to influence the pregnancy, one fetus in cephalic presentation, a pre-pregnant body mass index (BMI) ≤32, not smoking more than ten cigarettes per day, no prior operation on the uterus, no prior complicated deliveries, and spontaneous onset of labour between 36^+1^ and 41^+6^ weeks of gestation. Written informed consent was obtained from all study participants.

**Figure 1:**
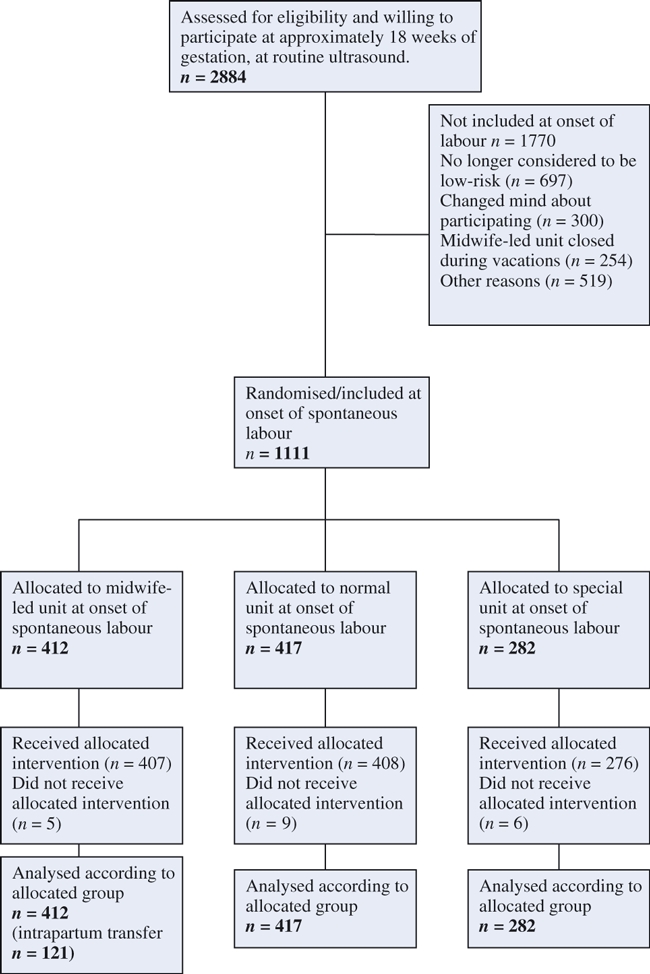
Flowchart of recruiting and inclusion process.

All 10 902 women who gave birth at the Østfold Hospital Trust during the study period were given written information on the trial when invited to ultrasound screening at 18 weeks of gestation. As the trial includes only healthy women, a certain number were excluded according to the inclusion criteria. Of the 2884 possible candidates assessed as being both eligible and willing to participate, 1773 did not meet the inclusion criteria by the time of onset of spontaneous labour for the following reasons: no longer considered to be at low risk because of pre-eclampsia, placenta praevia, intrauterine growth retardation, breech presentation, haemorrhage in third trimester, and pre- and post-term pregnancies and inductions (*n* = 697); changed their minds about participating (*n* = 300); the study was paused during summer and Christmas vacations because the MU was closed (*n* = 254); and for other reasons (*n* = 522). This led to a number of 1111 participants (Figure 1).

The randomisation process was performed through a digital randomisation database developed by the Clinical Research Unit at the University Hospital of North Norway. The midwife who administered the randomisation entered the women's name and checked for eligibility before receiving the randomisation number and unit from the database. Allocation was concealed and the randomisation stratified between primiparous (para 0) and multiparous (para 1+) women (Table 1). As the SU serves women with extended needs, their capacity to receive low-risk women is limited. Because of this, randomisation was pre-specified to allocate 37.5, 37.5 and 25.0% to the NU, MU and SU, respectively.

**Table 1:** Some basic characteristics of the participants

Variable	Midwife-led unit *n* = 282 (%)	Normal unit *n* = 417 (%)	Special unit *n* = 412 (%)
**Parity**			
Nulliparous(P0)	278 (67.5)	285 (68.3)	184 (65.2)
Multiparous(P+)	134 (32.5)	132 (31.7)	98 (35.4)
**Education**			
Primary school	20 (4.9)	25 (6.0)	23 (8.2)
High school	182 (44.2)	168 (40.3	112 (39.7)
College/university	202 (49.0)	218 (52.3)	139 (49.3)
Unknown	8 (1.9)	6 (1.4)	8 (2.8)
**Age**			
<25 years	103 (25.0)	100 (24.0)	64 (22.7)
25–35 years	263 (63.8)	270 (64.7)	181 (64.2)
>35 years	46 (11.2)	47 (11.3)	37 (13.1)
**Social status**			
Married	155 (37.6)	165 (39.6)	120 (42.6)
Cohabiting	236 (57.3)	229 (54.9)	152 (53.9)
Single	19 (4.6)	20 (4.8)	9 (3.2)
Unknown	2 (0.5)	3 (0.7)	1 (0.4)

### Documentation process

All data were registered by the midwife in charge in the electronic journal system of the department, partus (Clinsoft®), as is routine for all births. A midwife at each unit monitored the entries and was responsible for the documentation in connection with the trial. As a third and last documentation control, all the participants’ data were checked by a midwife not working at any of the three units.

### Statistical analysis

To detect a statistically significant reduction in operative delivery rate for low-risk women, from an estimated >10% in standard care units to approximately 5%, which is closer to the estimated rate in freestanding birth units, a power calculation was conducted. With a power of 80% and a probability of *P* < 0.05, one would have to include 1642 low-risk women. The inclusion process proceeded slower than expected, and unfortunately the funding was running out. Hence the inclusion stopped the first week of March 2010, including just 1111 participants in the trial. All data were analysed by the principle of ‘intention to treat’.

Analyses presenting differences between the three units were performed by chi-squared tests, and Pearson's two-sided asymptomatic significance level *P* values were calculated. The MU was set as the reference unit, and all primary and secondary outcomes of this unit were compared with the outcomes of the NU and SU. The statistician who performed the statistical analysis was blinded to the participants’ affiliation to the groups. Each result is presented with a risk ratio (RR) and a 95% confidence interval (95% CI). The analysis was conducted in statistical product and service solutions (SPSS) 17.

## Results

Of the 1111 participants in this trial, 67.2% were primiparous and 32.8% were multiparous. Table 1 shows the baseline characteristics of the participants.

### Mode of delivery

There was no statistically significant difference in mode of delivery between the three birth-care units (Table 2). At the MU, the total operative delivery rate was 16.3%, with 23.4% for primiparas. At the NU these figures were 18.0 and 25.6%, respectively, and at the SU the rates were 18.8 and 27.7% (Table 3).

**Table 2:** Relative risk (RR) assessments, with the MU set as the reference

Variable	MU vs NU RR (95% CI)	MU vs SU RR (95% CI)
Operative delivery	0.90 (0.67–1.22)	0.87 (0.62–1.20)
Operative vaginal delivery	0.85 (0.58–1.25)	0.98 (0.65–1.52)
caesarean section	1.01 (0.58–1.75)	0.71 (0.41–1.24)
Dystocia*	0.79 (0.65–0.96)	0.72 (0.59–0.89)
Oxytocin augmentation	0.71 (0.58–0.87)	0.69 (0.55–0.86)
Epidural	0.68 (0.51–0.90)	0.64 (0.47–0.86)
N_2_O	0.99 (0.90–1.09)	0.92 (0.83–1.02)
Acupuncture for pain relief	1.45 (1.25–1.69)	1.45 (1.22–1.73)
Postpartum haemorrhage >1000	0.79 (0.30–2.09	0.59 (0.20–1.41)
Episiotomy of all vaginal	0.85 (0.66–1.09)	0.78 (0.60–1.02)
Third-or fourth-degree tear of all vaginal deliveries	0.56 (0.19–1.66)	0.67 (0.20–2.28)
Apgar score <7 at 5 minutes	0.68 (0.19–2.37)	2.74 (0.31–24.37)
Metabolic acidocis**	0.78 (0.25–2.42)	1.10 (0.30–4.0)
Transfers to NICU***	1.25 (0.76–2.05)	1.15 (0.67–1.99)

* Midwife or doctor recorded labour dystocia, according to the hospital criteria.

** Metabolic acidosis: sample taken from umbilical cord showing arterial pH <7.05 and BE <−12 mmol/l.

*** Transfer of newborn to NICU within the first 2 hours postpartum.

**Table 3:** Birth outcome within the first 2 hours postpartum at all three birth care units

Variable	MU *n* = 412 (%)	NU *n* = 417 (%)	SU *n* = 282 (%)	*P*
**Mode of delivery**				
Total number of spontaneous deliveries	345 (84.0)	342 (82.0)	229 (81.0)	ns
Total number of operative deliveries	67 (16.0)	75 (18.0)	53 (18.8)	ns
Number of operative deliveries (P0)	65/278 (23.4)	73/285 (25.6)	51/184 (27.7)	ns
Number of operative deliveries (P+)	2/134 (1.5)	2/132 (1.5)	2/98 (2.0)	ns
Indication for operative delivery				
Labour dystocia	39 (58.2)	31 (41.3)	32 (60.4)	ns
Fetal distress	19 (28.4)	26 (34.7)	13 (24.5)	ns
Total number of operative vaginal deliveries	43 (10.0)	51 (12.0)	30 (11.0)	ns
Number of operative vaginal deliveries (P0)	42/278 (15.1)	49/285 (17.2)	29/184 (15.8)	ns
Number of operative vaginal deliveries (P+)	1/134 (0.7)	2/132 (0.7)	1/98 (1.0)	ns
Indication for operative vaginal delivery				
Labour dystocia	26 (60.5)	23 (45.0)	21 (70.0)	ns
Fetal distress	14 (32.6)	20 (39.2)	9 (30.0)	ns
Total number of caesarean sections	24 (6.0)	24 (6.0)	23 (8.0)	ns
Number of caesarean sections (P0)	23/278 (8.3)	24/285 (8.4)	22/184 (12.0)	ns
Number of caesarean sections (P+)	1/134 (0.7)	0/132 (0.0)	1/98 (1.0)	ns
Indication for caesarean section				
Labour dystocia	13 (54.2)	8 (33.3)	11 (47.8)	ns
Fetal distress	5 (20.8)	6 (25.0)	4 (17.4)	ns
Labour*	120 (29.0)	154 (37.0)	114 (40.0)	<0.01
**Oxytocin augmentation**	108 (26.2)	153 (36.7)	107 (38.0)	<0.01
**Epidural**	65 (16.0)	97 (23.0)	70 (25.0)	<0.01
**N**_**2**_**O**	270 (66.0)	275 (66.0)	201 (71.0)	ns
**Acupuncture for pain relief**	227 (55.0)	158 (38.0)	107 (38.0)	<0.001
**Postpartum haemhorrage**				
>1000 ml	7 (1.7)	9 (2.2)	9 (3.2)	ns
500–999 ml	33 (8.0)	38 (9.0)	36 (13.0)	ns
1000–1500 ml	4 (1.0)	6 (1.0)	3 (3.0)	ns
>1500 ml	3 (1.0)	3 (1.0)	6 (2.0)	ns
**Episiotomy, of all vaginal deliveries**	88/388 (23.0)	105/393 (27.0)	75/259 (29.0)	ns
**Third- or fourth-degree tear, all vaginal deliveries**	5 (1.0)	9 (2.0)	5 (2.0)	ns
Intrapartum transfer**	117 (28.0)			
**Apgar score <7 at 5 minutes**	4 (1.0)	6 (1.0)	1 (0.5)	ns
Metabolic acidocis***	5 (2.0)	7 (3.0)	4 (2.0)	ns
Transfers to NICU****	32 (8.0)	26 (6.0)	19 (7.0)	ns

* Midwife or doctor recorded labour dystocia, according to the hospitals criteria.

** Intrapartum transfer from the MU.

*** Metabolic acidosis: sample from umbilical cord showing arterial pH <7.05 and BE (Base Excess) <−12 mmol/l.

**** Transfer of newborn to NICU within the first 2 hours postpartum.

ns, not significant; P0, primiparous; P+, multiparous.

Of all 24 women delivered by caesarean section at the MU, 23 were primiparous. The main reason for the intervention was dystocia (54.2%). At the NU there were 24 caesarean sections, all of them among nulliparous women. Dystocia was the reason for 33.3% of the operations at this unit. At the SU, one multiparous and 22 nulliparous women were delivered by caesarean section, and the main reason given for this intervention was dystocia (47.8%) (Table 3).

Regarding operative vaginal delivery at the MU, 42 out of 43 deliveries were for primiparous women, and the main indication was dystocia. Of the women having an operative vaginal delivery at the NU, only two out of the 49 were multiparous, and the main reason for intervention was dystocia. At the SU, one of the 30 women who had an operative vaginal delivery was multiparous, and dystocia was the main indication for the interventions (Table 3).

#### Perineal outcome

There was no significant difference between the three groups concerning the number of episiotomies or the incidence of sphincter injuries (Table 2). An episiotomy was performed on 22.7, 26.7 and 29.0% of the women in the MU, NU and SU, respectively (Table 3). A sphincter injury occurred in 1.3, 2.3 and 1.9% of the vaginal deliveries at the MU, NU and SU, respectively (Table 3). Of the five women with a sphincter injury at the MU, none had an episiotomy, one had an operative vaginal delivery and four had spontaneous delivery. Of the nine women with sphincter injuries at the NU, four had both an episiotomy and operative vaginal delivery, one had spontaneous delivery with episiotomy and four had spontaneous delivery with no episiotomy. At the SU, five women had sphincter injuries: three had an operative vaginal delivery and episiotomy, one had an episiotomy and spontaneous delivery, and one had spontaneous delivery and no episiotomy.

#### Labour dystocia

Labour dystocia was evaluated and recorded by the midwives or the doctors. According to the hospital guidelines, dystocia is defined as progression of <1 cm dilatation of the cervix per hour in the active phase of the first stage (defined as 3–4 cm dilatation of the cervix and regular contractions until a cervix dilatation of 10 cm). Dystocia in the second stage is recorded if the expulsion phase lasts more than 60 minutes for both nulliparous and multiparous women. Dystocia in the second stage is also recorded if the second stage lasts longer than 2 hours for nulliparous women without epidural or multiparous women with epidural, or more than 3 hours for nulliparous women with epidural or more than 60 minutes for multiparous women without epidural. In the MU dystocia was recorded in 29.1% of the cases, which is a significantly lower rate than 36.9% in the NU (RR 0.79, 95% CI 0.65–0.96) and 40.4% in the SU (RR 0.72, 95% CI 0.59–0.89) (Table 2). Of all women allocated to the MU, 26.2% were given oxytocin infusion for augmentation of labour, which was significantly lower than 36.7% in the NU (RR 0.73, 95% CI 0.59–0.89) and 38.0% in the SU (RR 0.69, 95% CI 0.56–0.86) (Table 2). Labour dystocia was the main reason for all operative deliveries (Table 3).

The mean time for the active phase of the first stage was 4.9, 4.6 and 4.8 hours in the MU, NU and SU, respectively. The mean time for the expulsion phase of all vaginal deliveries was 40.4, 39.2 and 40.1 minutes in the MU, NU and SU, respectively.

#### Pain relief

Of all women randomised to the MU, 15.8% had an epidural, which is a significantly lower rate than 23.3% in the NU (RR 1.47, 95% CI 1.11–1.96) and 24.8% in the SU (RR 1.57, 95% CI 1.16–2.13). The women randomised to the MU had acupuncture in 55.1% of the cases, a significantly higher rate compared with those randomised to the NU (37.9%; RR 1.45, 95% CI 1.25–1.69) and SU (37.9%; RR 1.45, 95% CI 1.22–1.73) (Table 2).

#### Haemorrhage

There was no statistically significant difference in the rate of postpartum haemorrhage between the three units (Table 2). The vast majority of all participants had normal postpartum haemorrhage of 500 ml or less (MU 90.3%, NU 88.7% and SU 84.0%; Table 3). Of the 25 women with a haemorrhage of 1000 ml or more, 17 were caused by atonic postpartum haemorrhage (five operative vaginal deliveries, 11 spontaneous deliveries and one caesarean section), three were caused by a retained placenta (two operative vaginal deliveries and one spontaneous delivery) and for five women no indication was stated.

#### Neonatal outcomes

Neonatal outcomes were evaluated by Apgar score <7 at 5 minutes, metabolic acidosis and transfer to NICU within 2 hours of birth. An Apgar score <7 at 5 minutes was observed in 1.0, 1.4 and 0.4% of cases in the MU, NU and SU, respectively. An umbilical cord pH test was taken in 57.7, 68.8 and 77.3% of the cases in the MU, NU and SU, respectively. Metabolic acidosis was stated in 2.2, 2.8 and 2.0% in the MU, NU and SU, respectively. Transfers to the NICU were conducted in 7.8, 6.2 and 6.7% of the cases in the MU, NU and SU, respectively (Table 3). None of these outcomes showed a statistically significant difference between the units (Table 2).

#### Intrapartum transfer

Of the 412 women randomised to the MU, 117 (28.4%) were transferred intrapartum to a higher level of care, either to the NU or SU (Table 3). The reasons for transfer were need for pain relief (39.3%), stained amniotic fluid (18.8%), fetal distress (9.4%), labour dystocia (23.9%) and other reasons (8.5%). Mean dilatation of the cervix was 6.4 cm at the time of transfer; 51% were transferred with a cervix dilatation of <7 cm. Of all women transferred intrapartum, 61 (52.0%) had an operative delivery, and among these, 39.3% were delivered by caesarean section and 60.7% were delivered by operative vaginal delivery. Of those transferred for labour dystocia, 60.8% had an operative delivery and 39.2% had a spontaneous delivery.

## Discussion

In this randomised controlled trial three birth units within the same hospital were compared concerning birth care for low-risk women. The results when including 1111 participants showed no statistically significant differences in the total operative delivery rate, nor did it show differences in postpartum haemorrhage or neonatal outcomes between the three units.

Operative delivery rate is often used as a measure of the quality of birth care,^7^–^11^,^14^,^16^,^19^,^20^,^22^,^25^,^26^ but it is a subtle way of measuring quality, as it predicts poor quality if the operative delivery rate is low but gives a negative outcome, or if the rate is high without improving the outcome or even increasing the complications for the mother or newborn, yet it predicts good quality if performed when needed. Finding the right level or percentage of operative delivery will always be a challenge. Operative delivery rates differ between countries and institutions.

Data from the Medical Birth Registry of Norway (MBRN) from 2008 show an average total operative delivery rate for low-risk women of approximately 13.8%, but varies between institutions. It is worth noting that the birth population in Norway 2006–2009 consisted of 42.2% primiparous and 57.9% multiparous women.^21^ During the study period the Department of Obstetrics and Gynaecology at Østfold Hospital Trust had a high overall operative delivery rate (29.2%) compared with most hospitals in Norway.^21^ This fact is reflected in the high numbers of caesarean deliveries in this study in all three birth units. The overall high risk of having an operative delivery for low-risk nulliparas with no expressed preferences for level of birth care, leave birth attendants with the challenge of focusing on low-risk primiparous women, guiding them safely through their first labour.

Statistically significant differences were found for dystocia and augmentation of labour by oxytocin, and the use of epidural and acupuncture as pain relief. Moen et al.^27^ studied augmentation for all low-risk women in a retrospective study conducted at a large hospital in Norway. They found that low-risk women were given oxytocin in 39% of the cases (62% of the primiparous and 24% of the multiparous women), many of them without any documented indication. In a debate article in the *BMJ* in 2002, the authors state that medical interventions have become routine in normal childbirth, without evidence of effectiveness.^4^ This view is supported by others.^2^

A strength of this trial is the time for randomisation when comparing intrapartum birth care and birth outcome in low-risk birth units and standard care units. All participants were defined as low risk when entering the trial at onset of spontaneous labour, making sure that only those fulfilling the selection criteria were included. As far as we know no similar trial has been conducted in Europe during the last two decades.

A possible limitation of this trial is the fact that the number of women included was less than estimated by the power calculations, based on the primary outcome: operative delivery. This also might be the reason for the wide confidence interval for the primary outcome. However, the differences between the three units were so small that even if the total number of participants were included, it is considered unlikely that the differences would be significant. Only small non-significant differences in total operative delivery rate were found (total operative delivery rate, MU versus SU *P*= 0.57 and MU versus SU *P*= 0.44). There is a challenge in recruiting participants to studies like this because of the fact that women today often have their own preference for place of birth.^28^ This fact led to a longer recruiting period than expected in this trial.

## Conclusion

The operative delivery rate, the risk of having a postpartum haemorrhage of more than 1000 ml and the outcome for the newborn were not affected by the level of care for low-risk women without prelabour preferences for level of care. The participants randomised to the MU had a significantly higher chance of giving birth without interventions like augmentation by oxytocin or epidural analgesia. Further research is needed to determine the influence of women's own preference for birth care unit.

## References

[1] Jackson DJ, Lang JM, Swartz WH, Ganiats TG, Fullerton J, Ecker J (2003). Outcomes, safety, and resource utilization in a collaborative care birth center program compared with traditional physician-based perinatal care. Am J Public Health.

[2] Albers LL (2005). Overtreatment of normal childbirth in U.S. hospitals. Birth.

[3] Albers LL, Katz VL (1991). Birth setting for low-risk pregnancies. An analysis of the current literature. J Nurse Midwifery.

[4] Johanson R, Newburn M, Macfarlane A (2002). Has the medicalisation of childbirth gone too far?. BMJ.

[5] Mead MM, Kornbrot D (2004). The influence of maternity units’ intrapartum intervention rates and midwives’ risk perception for women suitable for midwifery-led care. Midwifery.

[6] Kenyon S, Ullman R, Mori R, Whittle M (2007). Care of healthy women and their babies during childbirth: summary of NICE guidance. BMJ.

[7] Rooks JP, Ernst EK (1990). Outcomes of care in birth centers. Birth.

[8] Schmidt N, Abelsen B, Oian P (2002). Deliveries in maternity homes in Norway: results from a 2-year prospective study. Acta Obstet Gynecol Scand.

[9] Walsh D, Downe SM (2004). Outcomes of free-standing, midwife-led birth centers: a structured review. Birth.

[10] Waldenstrom U, Nilsson CA (1997). A randomized controlled study of birth center care versus standard maternity care: effects on women's health. Birth.

[11] Hodnett ED, Downe S, Walsh D, Weston J (2010). Alternative versus conventional institutional settings for birth. Cochrane Database Syst Rev.

[12] Gottvall K, Grunewald C, Waldenstrom U (2004). Safety of birth centre care: perinatal mortality over a 10-year period. BJOG.

[13] Stewart M, McCandlish R, Henderson J, Brocklehurst P (2004). Report of a structured review of birth centre outcomes [homepage on the Internet]. http://www.npeu.ox.ac.uk/birthcentrereview.

[14] Hatem M, Sandall J, Devane D, Soltani H, Gates S (2008). Midwife-led versus other models of care for childbearing women. Cochrane Database Syst Rev.

[15] Byrne JP, Crowther CA, Moss JR (2000). A randomised controlled trial comparing birthing centre care with delivery suite care in Adelaide, Australia. Aust N Z J Obstet Gynaecol.

[16] Harvey S, Jarrell J, Brant R, Stainton C, Rach D (1996). A randomized, controlled trial of nurse-midwifery care. Birth.

[17] Hundley VA, Cruickshank FM, Lang GD, Glazener CM, Milne JM, Turner M (1994). Midwife managed delivery unit: a randomised controlled comparison with consultant led care. BMJ.

[18] MacVicar J, Dobbie G, Owen-Johnstone L, Jagger C, Hopkins M, Kennedy J (1993). Simulated home delivery in hospital: a randomised controlled trial. Br J Obstet Gynaecol.

[19] Chambliss LR, Daly C, Medearis AL, Ames M, Kayne M, Paul R (1992). The role of selection bias in comparing cesarean birth rates between physician and midwifery management. Obstet Gynecol.

[20] Law YY, Lam KY (1999). A randomized controlled trial comparing midwife-managed care and obstetrician-managed care for women assessed to be at low risk in the initial intrapartum period. J Obstet Gynaecol Res.

[21] Quality indicators—Medical Birth Registry of Norway. http://mfr.no/.

[22] Holt J, Vold IN, Backe B, Johansen MV, Øian P (2001). Child births in a modified midwife managed unit: selection and transfer according to intended place of delivery. Acta Obstet Gynecol Scand.

[23] (2000). Recommendation from the Norwegian Social Committee (Innstilling fra Sosialkomiteen om akuttmedisinsk beredskap. http://www.stortinget.no/no/Saker-og-publikasjoner/Innstillinger/Stortinget/2000-2001/inns-200001-300/1/.

[24] Siggaard-Andersen O (1971). An acid-base chart for arterial blood with normal and pathophysiological reference areas. Scand J Clin Lab Invest.

[25] Eide BI, Nilsen AB, Rasmussen S (2009). Births in two different delivery units in the same clinic—a prospective study of healthy primiparous women. BMC Pregnancy Childbirth.

[26] Nesheim BI, Eskild A, Gjessing L (2010). Does allocation of low risk parturient women to a separate maternity unit decrease the risk of emergency cesarean section?. Acta Obstet Gynecol Scand.

[27] Moen MS, Holmen M, Tollefsrud S, Rolland R (2005). Low-risk pregnant women in an obstetric department-how do they give birth?. Tidsskr Nor Laegeforen.

[28] Hendrix M, Van Horck M, Moreta D, Nieman F, Nieuwenhuijze M, Severens J (2009). Why women do not accept randomisation for place of birth: feasibility of a RCT in the Netherlands. BJOG.

